# Digital Pathology During the COVID-19 Outbreak in Italy: Survey Study

**DOI:** 10.2196/24266

**Published:** 2021-02-22

**Authors:** Simone Giaretto, Salvatore Lorenzo Renne, Daoud Rahal, Paola Bossi, Piergiuseppe Colombo, Paola Spaggiari, Sofia Manara, Mauro Sollai, Barbara Fiamengo, Tatiana Brambilla, Bethania Fernandes, Stefania Rao, Abubaker Elamin, Marina Valeri, Camilla De Carlo, Vincenzo Belsito, Cesare Lancellotti, Miriam Cieri, Angelo Cagini, Luigi Terracciano, Massimo Roncalli, Luca Di Tommaso

**Affiliations:** 1 Department of Biomedical Sciences Humanitas University Pieve Emanuele (MI) Italy; 2 Department of Pathology Humanitas Clinical and Research Center – IRCCS Rozzano (MI) Italy

**Keywords:** COVID19, digital pathology, Bayesian data analysis, probabilistic modeling

## Abstract

**Background:**

Transition to digital pathology usually takes months or years to be completed. We were familiarizing ourselves with digital pathology solutions at the time when the COVID-19 outbreak forced us to embark on an abrupt transition to digital pathology.

**Objective:**

The aim of this study was to quantitatively describe how the abrupt transition to digital pathology might affect the quality of diagnoses, model possible causes by probabilistic modeling, and qualitatively gauge the perception of this abrupt transition.

**Methods:**

A total of 17 pathologists and residents participated in this study; these participants reviewed 25 additional test cases from the archives and completed a final psychologic survey. For each case, participants performed several different diagnostic tasks, and their results were recorded and compared with the original diagnoses performed using the gold standard method (ie, conventional microscopy). We performed Bayesian data analysis with probabilistic modeling.

**Results:**

The overall analysis, comprising 1345 different items, resulted in a 9% (117/1345) error rate in using digital slides. The task of differentiating a neoplastic process from a nonneoplastic one accounted for an error rate of 10.7% (42/392), whereas the distinction of a malignant process from a benign one accounted for an error rate of 4.2% (11/258). Apart from residents, senior pathologists generated most discrepancies (7.9%, 13/164). Our model showed that these differences among career levels persisted even after adjusting for other factors.

**Conclusions:**

Our findings are in line with previous findings, emphasizing that the duration of transition (ie, lengthy or abrupt) might not influence the diagnostic performance. Moreover, our findings highlight that senior pathologists may be limited by a digital gap, which may negatively affect their performance with digital pathology. These results can guide the process of digital transition in the field of pathology.

## Introduction

Digital pathology (DP) intends to use computer workstations and digital whole slide imaging to diagnose a pathological process [1-4]. A complete transition from classical to digital pathology is usually a “soft” procedure, taking months or even years to be completed [4-9]. We planned a digitalization of our department, and we were testing several technical aspects related to digital transition. By February 2020, most of our staff pathologists and residents had used digital whole slide imaging for educational or scientific purposes, but the situation radically changed in March 2020. With the COVID-19 pandemic and the subsequent guidelines adopted by the Italian national government and the medical direction of our hospital, we were forced to reduce the presence of staff in the laboratory. Taking advantage of the ongoing digitalization, we decided to adopt DP to sustain smart work.

Most of the reported discordances between diagnoses in DP and those by the gold standard (ie, evaluation of a glass slide under a microscope) are less than 10% [10], and none of these reports were made under an abrupt transition in diagnostic approach. These discrepancies could be attributed to several factors that could be pathologist dependent (eg, career level or individual performance) or pathologist independent (eg, specimen type or the task to be undertaken during the diagnostic procedure). Discerning the relative effect of these features (that could be really small)—even in a carefully designed experimental setting—might be challenging. Probabilistic modeling (and Bayesian data analysis, in general) allows the detection of small effects [11-13]. Moreover, the employment of multilevel hierarchical modeling permits the transfer of shared information among data clusters, resulting in balanced regularization; thus, it reduces overfitting and improves the out-of-sample predictive performance [11,14-18].

In this study, we aimed to (1) quantitatively describe how abrupt transition to DP might affect the quality of diagnosis, (2) model the possible causes via probabilistic modeling, and (3) qualitatively gauge the perception of this abrupt transition.

## Methods

A detailed description of the study methods is described in Multimedia Appendix 1 [15,16,19-24].

### Ethics Approval

No ethics approval was required for this study. The study participants (ie, pathologists and residents) agreed to—and coauthored—the study.

### Study Participants

This study involved 17 participants who were divided into the following 4 groups or career levels, based on their pathology experience: (1) senior (pathologists with >20 years of experience, n=2), (2) expert (pathologists with 10-20 years of experience, n=5), (3) junior (pathologists with <10 years of experience, n=6), and (4) resident (1st year, n=1; 2nd year, n=3). Each of the 17 participants evaluated 25 digital cases, with a total of 425 digital images examined in the study. Overall, 1445 questions were examined (ie, 85 questions per participant) in the study.

### Study Design

In addition to their own diagnostic tasks, which were not considered in this study, the pathologists and residents received (1) a set of digital cases within the area of general surgical pathology, (2) specific questions to be addressed while reviewing the cases, and (3) a survey about their digital experience.

### Sets of Digital Cases

We set up 5 sets of digital cases representing 3 different specialties (breast: n=2; urology: n=1; and gastrointestinal: n=2) and assigned them to each study participant. Each test comprised 5 cases, represented by one or more slides of a single case that was previously diagnosed using conventional microscopy by the referral pathologist at our institution. The information reported about the original diagnosis was considered as the gold standard. To cover a spectrum of conditions overlapping the routine situation, we considered biopsy and surgical specimens (specimen type). Cases were digitalized using the Aperio AT2 scanner (Leica Biosystems) and visualized using the WebViewer APERIO ImageScope (version 12.1). The slides used for the tests were from 8 nontumoral and 17 tumoral cases. Of the tumoral cases, 7 tumors were benign and 10 were malignant; all malignant tumors were infiltrative and equally distributed between grade 2 and grade 3; 14 cases were biopsy and 11 were surgical.

### Study Questionnaire

Participants answered (all or some) of the following questions (ie, categories of diagnostic task), for each case: (1) Is it neoplastic or negative for neoplasia? (2) Is it a malignant (in situ or infiltrative) or a benign neoplasia? (3) What is the histopathological diagnosis? (4) What is the histotype of the lesion? (5) What is the grade of the lesion? Questions 1 and 3 were answered for all cases, question 2 was answered only for neoplastic lesions, and questions 4 and 5 were answered for malignant neoplasms.

### Statistical Analysis

To model data clusters, we used a varying effects, multilevel (hierarchical) model [14-16]. The rate of wrong answers (*W_i_*) was modeled as a Bernoulli distribution:

*W_i_* ∼ *Binomial* ( 1, *p_i_* )

For each pathologist (PID), their career level (LEVEL), the specific diagnostic question (CATEGORY), the specimen type (SPECIMEN), and the subspecialty of the case (SPECIALTY), we used the logit link function and modeled the varying intercepts as follows:


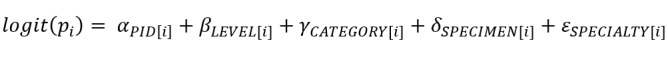


The prior distribution for the intercepts and SD values were as follows:

*α_j_* ∼ *Normal* ( 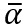 , σ_α_ ), for *j* = 1..17


*β_j_* ∼ *Normal* ( 0 , σ_β_ ), for *j* = 1..4

*γ_j_* ∼ *Normal* ( 0 , σ_γ_ ), for *j* = 1..5

*δ_j_* ∼ *Normal* ( 0 , σ_δ_ ), for *j* = 1..2

*ε_j_* ∼ *Normal* ( 0 , σ_ε_ ), for *j* = 1..3

σ_β_ ∼ *Exponential* ( 1 )

σ_γ_ ∼ *Exponential* ( 1 )

σ_δ_ ∼ *Exponential* ( 1 )

σ_ε_ ∼ *Exponential* ( 1 )

The hyperpriors for the hyperparameters average pathologist 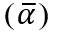 and σ*_α_* were set as follows:


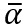
 ∼ *Normal* ( 0, 1.5 )



σ_α_ ∼ *Exponential* ( 1 )

The SD value for 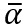 was set at 1.5 since it produces a flat (weakly regularizing) prior after logit transformation [16,18]; moreover, we used an exponential distribution to model SD, because this assumes the least, for maximum entropy reasons [16,25-28], given the fact that σ is a nonnegative continuous parameter. To assess the validity of priors, we run prior predictive simulation of the model [16,29,30] (see Table S1 in Multimedia Appendix 1, and Multimedia Appendices 2 and 3). To limit divergent transitions, we reparametrized the models with a noncentered equivalent form [31,32]. Models were fit using Stan (a probabilistic programming language) and R [33,34]. Full anonymized data and custom code can be found in the public repository SmartCovid hosted on Github [35].

### Study Survey

The survey was inspired by previous published works [36-38]. Briefly, the survey included 17 questions in a randomized order for all the pathologists, covering 3 fields: (1) attitude towards DP, (2) confidence in using DP solutions, and (3) satisfaction with DP. The survey was sent at the end of the digital experience. Pathologists were requested to answer the questions using a Likert scale, with scores ranging from 1 (strongly disagree) to 5 (strongly agree). The results were reported as the proportion of pathologists who assigned each single value of the Likert scale.

## Results

### Quantitative Description

**Figure 1:**
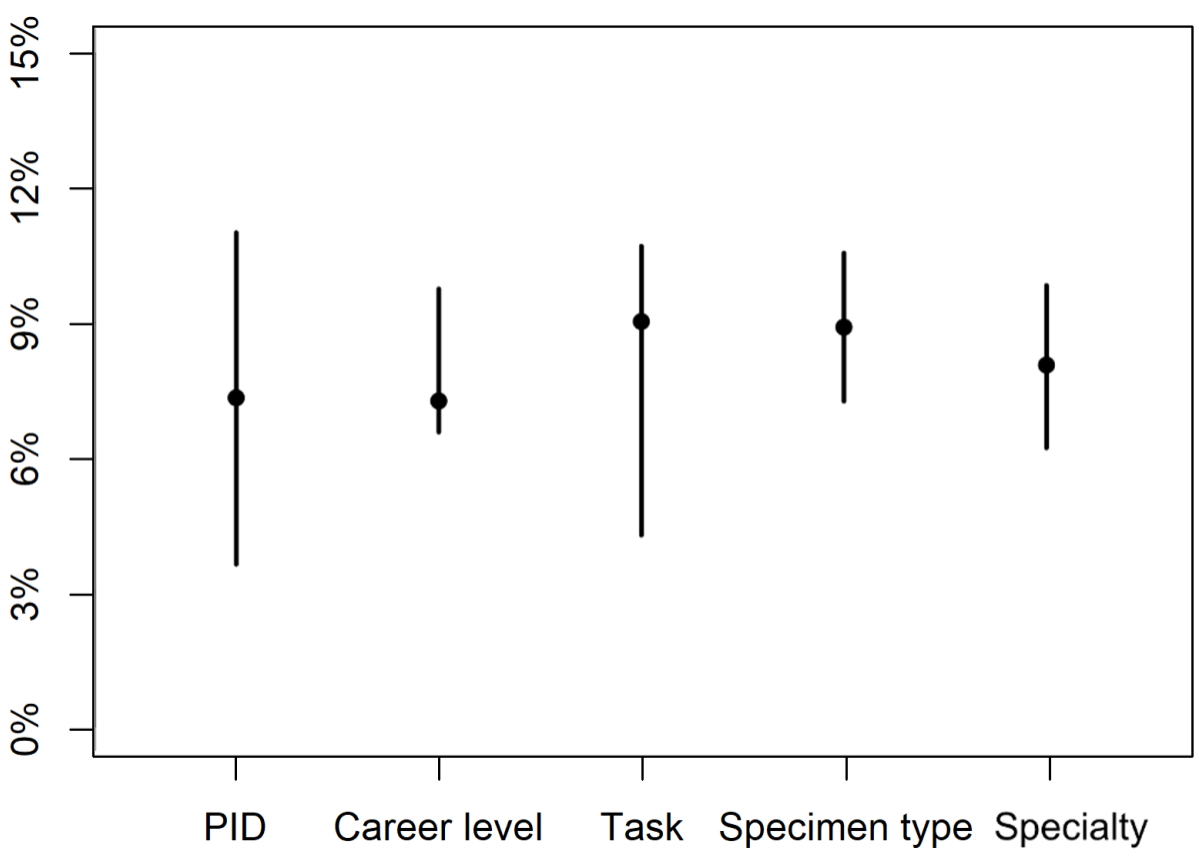
Error rates among different categories. This dot-bar plot depicts the median (IQR) values of error rates among different categories. The error rates showed the widest IQR among individual pathologists (PID), whereas the least IQR was noted for the career level and the specimen type (biopsy vs surgical).

The pathologists answered 1345 of the total 1445 questions (100 missing answers in total), of which 1228 (91.30%) corresponded to the original diagnoses and were considered correct. Table 1 depicts the errors among each group of the 5 different categories recorded, and Figure 1 highlights the median (IQR) values of those categories. Considerable variation was observed among the performances of each pathologist, ranging from an error rate of 0.01 (1/67, Pathologist #4) to 0.32 (26/81, Pathologist #13), with a collective median error of 0.07 (IQR 0.04-0.11). This performance variation was tapered once the same data were considered after filtering among the different career levels, yielding the same median of 0.07, but a narrower IQR of 0.07-0.10. Moreover, some diagnostic tasks were more error prone than others; for instance, histotyping of the lesions had a very low rate of errors 0.01 (2/160), whereas grading was a more error-prone task with an error rate of 0.18 (27/147). The specimen type also resulted in different error rates, with surgical specimens easier to diagnose, with an error rate of 0.06 (40/716), than biopsy specimens, with a 2-fold error rate at 0.12 (77/629).

**Table 1:** Proportion of errors among different groups.

Group		Number of tasks performed	Number of errors	Error rate
**Pathologist ID**				
	P1	84	5	0.06
	P2	78	4	0.05
	P3	82	7	0.09
	P4	67	1	0.01
	P5	82	7	0.09
	P6	82	6	0.07
	P7	83	2	0.02
	P8	84	3	0.04
	P9	82	5	0.06
	P10	83	3	0.04
	P11	82	9	0.11
	P12	83	3	0.04
	P13	81	26	0.32
	P14	64	9	0.14
	P15	84	12	0.14
	P16	79	9	0.11
	P17	65	6	0.09
**Career level**				
	Resident	310	47	0.15
	Junior	460	30	0.07
	Expert	411	27	0.07
	Senior	164	13	0.08
**Category of the diagnostic task**				
	Neoplasia?	392	42	0.11
	Malignant/benign?	258	11	0.04
	Histopathological diagnosis?	388	35	0.09
	Histotype?	160	2	0.01
	Grade?	147	27	0.18
**Specimen type**				
	Surgery	716	40	0.06
	Biopsy	629	77	0.12
**Case subspecialty**				
	Breast	550	64	0.12
	Gastrointestinal	497	40	0.08
	Urology	298	13	0.04
Total		1345	117	0.09

**Figure 2:**
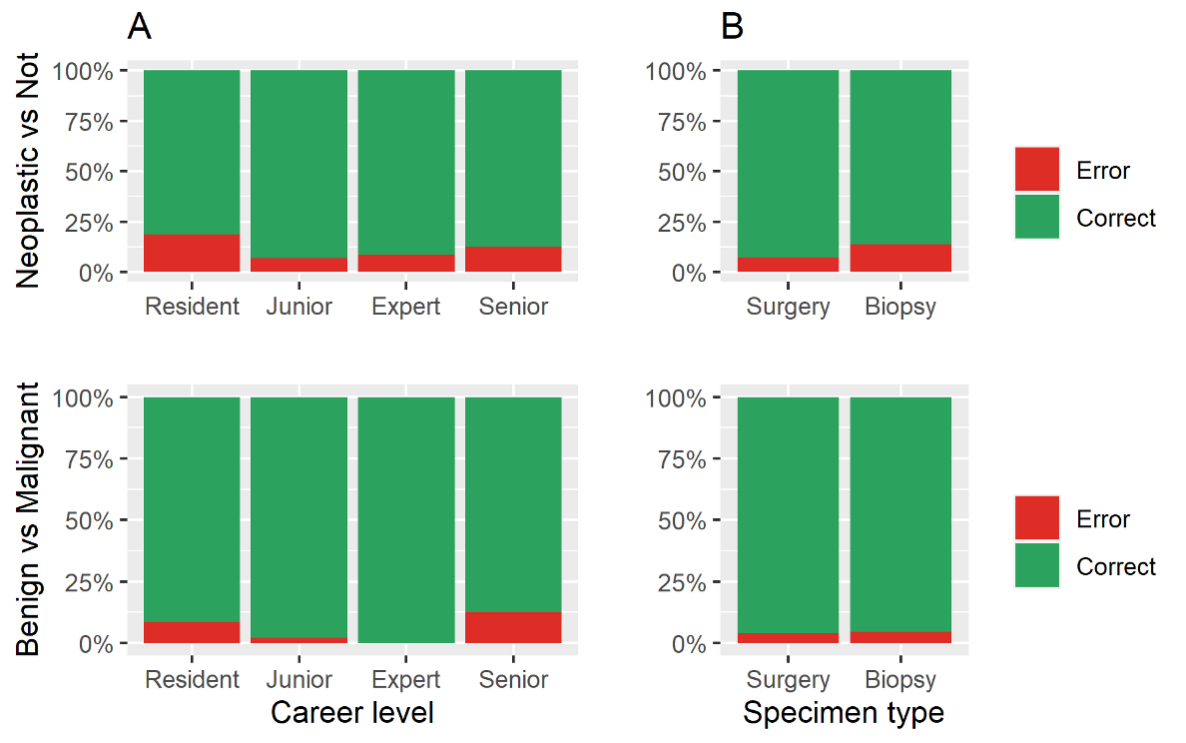
Raw proportion of errors across (A) career levels and (B) specimen types in performing two important tasks: differentiation between neoplastic and nonneoplastic processes and between malignant and benign tumors.

Differences in error rates for two important tasks—differentiation between neoplastic and nonneoplastic processes and that between benign and malignant neoplastic processes—were observed among pathologists at different career levels and for different specimen types. The same error profile was observed across career levels, although the former task had a higher error rate (Figure 2A). However, even though the differentiation of a neoplastic process from a nonneoplastic one might be more challenging on a biopsy specimen, the distinction between a benign and malignant neoplasm was done with the same error rate regardless of the specimen type (Figure 2B). Differences in the prevalence of errors among individual pathologists and those at different career levels, as well as across diagnostic tasks, specimen type, and case subspecialty, are further highlighted in Multimedia Appendices 4 and 5.

### Prediction of Average Pathologist Performance

**Figure 3:**
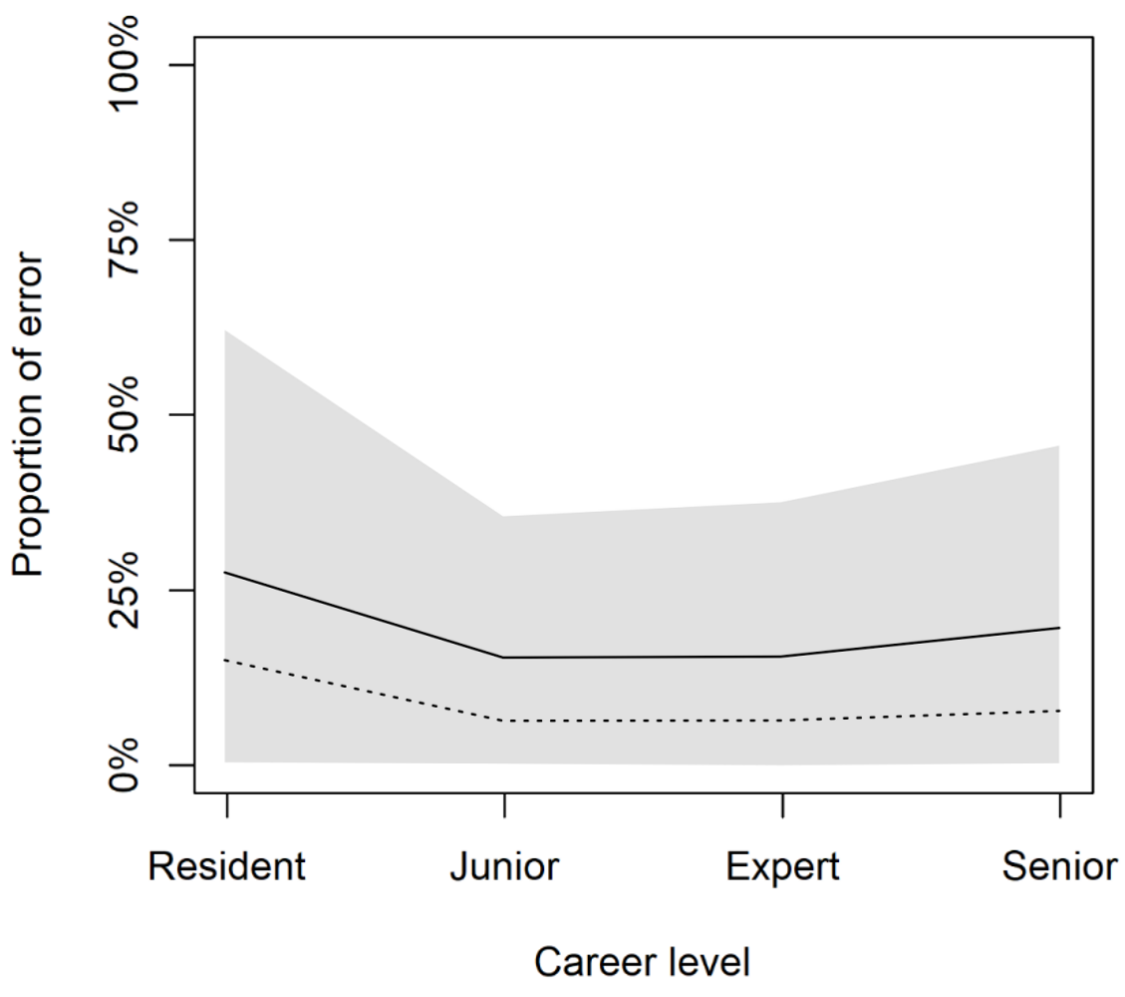
Prediction of average pathologist performance. Pathologists of intermediate levels of career perform better on average. The graph depicts the posterior predictive distributions for the multilevel model. Solid lines represent posterior mean values; shaded regions represent 89% high-posterior density interval; and dashed lines represent raw data.

Diagnostics of the model’s fit are shown in Multimedia Appendices 6, 7, and 8. The analysis reported a good overall performance: the average pathologist 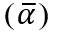 showed a negative mean coefficient of -1.8 with most of the posterior probability mass below 0 (given the model structure, positive values reflect the probability of making errors; Table S2 in Multimedia Appendix 1). The pathologists’ individual performances and their career levels were the variables that showed less variance in predicting the error rate, whereas the specimen type, case subspecialty, and the particular type of task collectively showed more variance (Multimedia Appendix 9). Hence, we simulated the performance of an average pathologist at different career levels; this prediction shows better performance among pathologists at intermediate career levels of career (Figure 3).

### Survey Results

**Figure 4:**
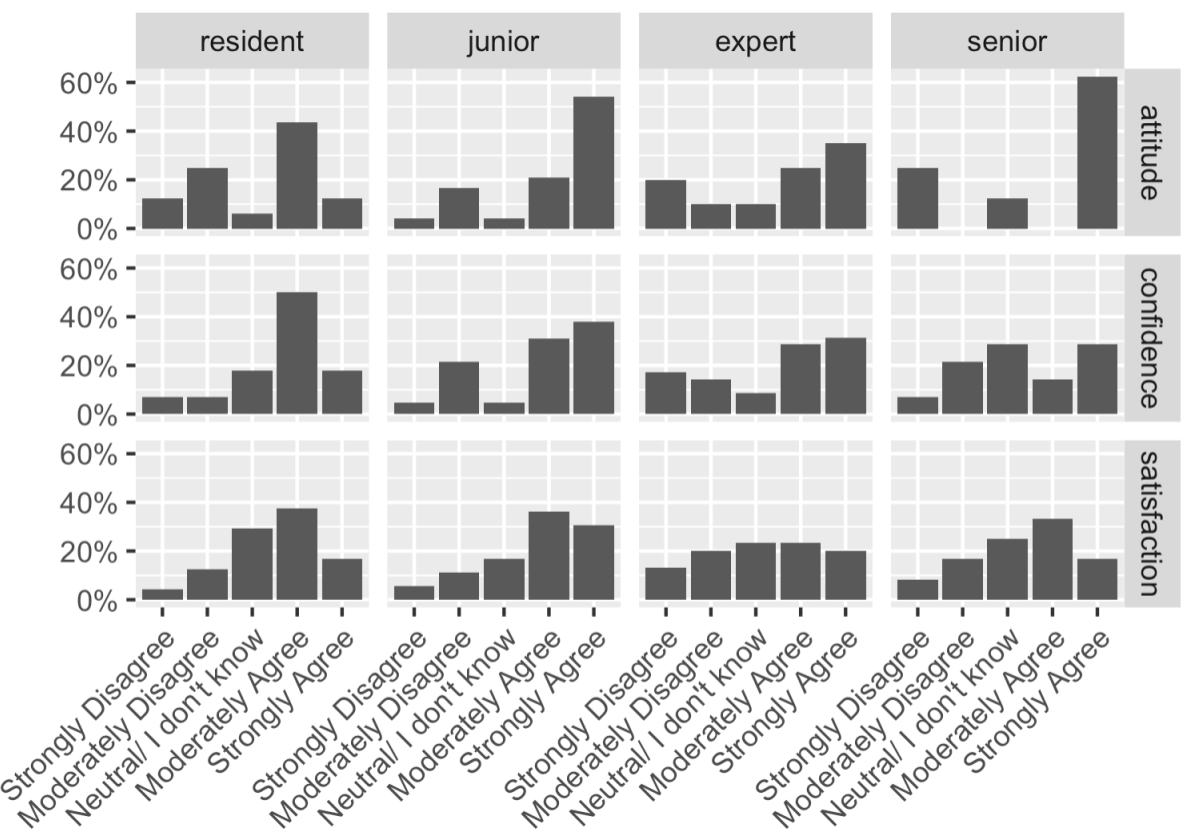
Overview of the psychological aspect of the study. This series of graphs summarize the results of the survey conducted among pathologists at different career levels (residents, junior, expert, and senior) to evaluate their attitudes toward, confidence in, and satisfaction with digital pathology solutions.

Most pathologists reported a very good score (ie, 4 or 5 indicating they “moderately agree” and “strongly agree,” respectively) for their attitude toward DP (44/68, 64%), confidence in DP (75/119, 63%), and satisfaction with DP (56/102, 54.9%). A detailed analysis of these parameters showed that the residents reported the highest value for confidence, junior pathologists reported the highest values for attitude and satisfaction, whereas expert and senior pathologists reported relatively lower levels of confidence in and satisfaction with DP (Figure 4).

## Discussion

### Principal Findings

Our study showed an overall discordant rate of 9% among diagnoses performed using digital slides and those performed using the gold standard (ie, conventional microscopy). However, when we considered the different diagnostic tasks, this rate dropped to less than 5% in the category “benign versus malignant tumor”, which is probably the most clinically impacting category among the other diagnostic tasks. A systematic review of 38 pertinent studies published before 2015 reported a 7.6% overall discordance rate between digital and glass slide diagnoses. Among these studies, 17 studies reported a discordant rate higher than 5%, and 8 reported a discordant rate higher than 15% [39]. A later reanalysis of the same series fixed the overall discordance rate to 4% and major discrepancies to 1% [40]. A more recent review, covering studies published until 2018, reported a disagreement ranging from 1.7% to 13% [10]. Two multicentric, randomized, non-inferiority studies reported major discordant rates of 4.9% [41] and 3.6% [42] between diagnoses done by digital and glass slides. Furthermore, a study from a single, large academic center reported an overall diagnostic equivalency of 99.3% [43]. The same group was also the first to report about the use of DP during COVID-19 with an overall concordance of 98.8% [44]. Thus, despite our challenging approach to DP, the diagnostic performance we recorded was consistent with previous reports—a result that further supports the transition to DP.

In our study, a high proportion of errors was generated in small biopsy specimen type (12.2%) and diagnostic tasks involving tumor grading (23%). These results are consistent with those of the review by Williams et al [40]. The latter showed that 21% of all errors concerned grading or histotyping of malignant lesions, whereas 10% of the errors could be ascribed to the inability to find the target.

Moreover, recent studies have consistently reported high, intermediate, and low discordant rates for bladder, breast, and gastrointestinal tract specimens, respectively [41,42]—a finding suggesting intrinsic difficulties of specific areas. In contrast, we observed 4%, 8%, and 12% of discrepancies for urology, gastrointestinal tract, and breast specimens. This result could be attributed to a nonrandom selection of the cases and might represent a study limitation, biasing the value of the coefficients of specific parameters of the case subspecialty, similar to those of diagnostic tasks and the specimen type. However, these characteristics were excluded in the posterior predictive simulation, which was intended to represent how the different career levels might impact the pathologists’ performance, after adjusting for all other factors.

As compared by the study by Hanna et al [44], our readiness to undertake digital diagnostic tasks was far from being mature in March 2020, and this study was specifically designed to identify and illustrate the effects of such a sudden adoption of DP—something that had never been investigated before. Our results suggest that this abrupt transition might not influence the adoption of and performance with DP. However, different factors seem to be involved. In particular, data concerning major discrepancies between diagnoses using DP and gold standard methods disclosed an interesting feature. Both in the distinction of neoplastic versus non-neoplastic lesions and of benign versus malignant tumors, the worst results obtained were among residents and senior pathologists–2 contrasting categories in terms of pathologists’ working experience. Therefore, these survey results might suggest an explanation to this paradoxical result: senior pathologists felt ready to diagnose a pathological process using a digital approach (ie, positive attitude) but were less prepared to use digital devices (ie, low confidence). Residents, in turn, had a high predisposition to using a digital device (ie, high confidence) but also had some concerns about diagnosis of a pathological process (ie, poor attitude). The hypothesis that senior pathologists were limited by a digital gap was supported by another finding: once they decided a lesion was malignant, they demonstrated the best performance with regard to tumor grading. By contrast, residents made several errors, likely due to their limited working experience. Lastly, even if expert pathologists showed a good diagnostic performance, they had the lowest level of satisfaction in DP. This result suggests that DP can be adopted rapidly for practical purposes. However, it also highlights a critical point of the process that needs to be addressed, possibly with adequate training or user-friendly equipment, and warrants further investigations.

### Conclusions

Our study describes how the abrupt transition to DP affected the quality of diagnoses and qualitatively gauged the psychological aspects of this abrupt transition. Moreover, our study model highlighted the potential causes for these challenges and might inform what could be expected in other laboratories. In conclusion, the exceptional conditions dictated by the COVID-19 pandemic highlighted that DP could be adopted safely for diagnostic purposes by any skilled pathologist, even abruptly.

## Appendix

Multimedia Appendix 1 Supplementary materials and methods.

Multimedia Appendix 2 Coefficients of model parameters from the prior predictive simulation.

Multimedia Appendix 3 Simulation from the prior. This figure shows the meaning of the priors (ie, what the model thinks before it sees the data).

Multimedia Appendix 4 Proportion of errors among individual pathologists. Upper left panel shows the overall error rates. Upper right panel shows the error rates among different diagnostic tasks. Lower left panel shows the error rate among different specimen types. Lower right panel highlights the different error rates among different case subspecialties. GI: gastrointestinal, Uro: urology.

Multimedia Appendix 5 Proportion of errors among different career levels. Upper left panel shows the overall error rates. Upper right panel shows the error rates among the different diagnostic tasks. Lower left panel shows the error rate among different specimen types. Lower right panel highlights the different error rates among different case subspecialties. GI: gastrointestinal, Uro: urology.

Multimedia Appendix 6 Traceplot of the model fit - part A.

Multimedia Appendix 7 Traceplot of the model fit - part B.

Multimedia Appendix 8 Traceplot of the model fit - part C.

Multimedia Appendix 9 Model coefficients. Graphical representation of the coefficients for the model parameters conditional on the data. The lowest box depicts the coefficients for the hyper-parameter α¯ (alpha_bar) and the variances – the σ (sigma_a, b, [...] e) – of the categories of clusters modeled. All other boxes depict the distributions of the mean value for each element of the category considered. From top to bottom: the first box depicts the parameters of the pathologists’ performance; the second, the parameters regarding the career level; the third, the diagnostic category analyzed; the fourth, the specimen type; and the fifth, the case subspecialty. Interpretation of the model at the parameter level is not possible because they combine in a very complicated way: prediction (ie, see how the model behave on the outcome scale, Figure 4 in the manuscript) is the only practical way to understand what the model “thinks”.

## Abbreviations

DP: digital pathology
