# Supplementary material for: Digital Pathology During the COVID-19 Outbreak in Italy: Survey Study
Source: J Med Internet Res. 2021 Feb 22;23(2):e24266. doi: 10.2196/24266 (PMC7901595; doi:10.2196/24266)
Supplement: Multimedia Appendix 1 [file jmir_v23i2e24266_app1.docx]

# SUPPLEMENTARY

***Supplementary Materials and methods***

***Criteria used to select cases***

*Cases selection:* the PI (LDT) and a referral pathologist (the pathologist in charge, at the Institution, for a specific area, for example breast) identified cases falling within the area of general surgical pathology (breast cancer and its differential diagnosis; prostate cancer and its differential diagnosis; etc) diagnosed between 01-01-2020 and 01-03-2020. *Slide selection:* the PI (LDT) and a referral pathologist identified, among each case, the single H/E which best represented the pathologic process and could be confidently diagnosed even without the aid of clinical data, immunohistochemistry or molecular biology.

***List of cases***

Once selected according to the above-mentioned criteria, cases were grouped into five sets of test as detailed below; the tests were submitted to pathologists by the end of each week of smart work, starting from the second week (2nd week-1st test; 3rd week-2nd test; etc) and for 5 consecutive weeks in the following order:

*BREAST TEST 1*: radial scar (BR1.1); infiltrative carcinoma NST (BR 1.2); ductal hyperplasia (BR 1.3); infiltrative carcinoma, lobular type (BR 1.4); fibroadenoma (BR 1.5). *BREAST TEST 2*: papillary lesion (BR 2.1); nodal metastasis by NST carcinoma (BR 2.2); fibroadenoma (BR 2.3); fibro-epithelial lesion (BR 2.4); infiltrative carcinoma NST (BR 2.5). *URO TEST*: benign prostatic hyperplasia (URO 1.1); infiltrative adenocarcinoma of the prostate, acinar type (URO 1.2); benign prostatic hyperplasia (URO 1.3); infiltrative urothelial carcinoma of the bladder (URO 1.4); surrenal metastasis by a clear cell renal carcinoma (URO 1.5). *GI TEST 1*: acute appendicitis (GI 1.1); hyperplastic polyp (GI 1.2); tubulo-villous adenoma (GI 1.3); infiltrative adenocarcinoma of the stomach, discohesive type (GI 1.4); tubulo-villous adenoma (GI 1.5). *GI TEST 2*: tubular adenoma (GI 2.1); infiltrative adenocarcinoma of the colon (GI 2.2); granulation tissue (GI 2.3); hepatic metastasis by a colorectal carcinoma (GI 2.4); chronic cholecystitis and reactive lymph-node (GI 2.5).

***Diagnostic criteria and terminology***

Pathologists received an excel file with each set of cases; questions 1 and 2 were pre-filled (tumor/not tumor; malignant/not malignant) and pathologists should leave just one answer; questions 3, 4 and 5 were left blank and pathologists could fill them with any text; if present, discrepancies in terminology were discusses and fixed by the PI and the pathologist involved.

***Questionnaire***

*Attitude with DP (how you think or feel about DP)*

1) I knew about digital pathology before the covid19 situation

2) I had already practiced digital pathology before the covid19 situation

3) When I learned that I would use digital pathology as a smart working tool I was skeptical*

4) In the absence of a strong stimulus I would not have let myself be involved in using digital pathology*

*Confidence with DP (the belief that you are able to use DP well or be successful with DP)*

1) I found the digital slide viewing program intuitive to use

2) I did not found difficulties to assign a diagnostic category to the digital slide (tumor vs. non-tumor)

3) I found no difficulty in assigning a histopathological diagnosis to the digital slide

4) I think the mouse is a functional tool for the panning of the digital slide

5) I am interested in any other strumental peripherics other than the mouse to view the digital slide

6) I have an unclear management of the slides of each individual patient with the web viewer respect to the use of the physical tray*

7) I did not find difficulties in terms of overall vision and handling, to examine the digital slides

*Satisfaction after DP (a good feeling because you have done well with DP)*

1) After this experience I still have doubts about digital pathology as a smart working tool*

2) After this experience I am more interested in digital pathology

3) I believe that the time required to perform in a satisfactory way with digital pathology is excessive*

4) After this experience I feel comfortable in the diagnosis using digital pathology

5) I felt less satisfied at the end of the view of the case using digital pathology*

6) I am satisfied how I worked using digital pathology

*for these questions the value of agreement was overturned.

***Criteria to fill the questionnaire***

Mark under each question a number from 1 to 5 which mean respectively: 1 completely disagree; 2 moderately disagree; 3 neutral / I don't know; 4 moderately agree; 5 completely agree.

***Scanner, viewer and connection***

The Aperio AT2 DX system (Leica Biosystems, Inc., Vista, California) used during this study included a scanner with a 0.75 numerical aperture 320 objective in a line scan format with multipoint autofocusing. The ImageScope browser software (Leica Biosystems, Inc.) includes tools for zoom, rotation, and navigation, including a navigation history pane that indicates unexamined areas of each WSI. The pathologists evaluated digital slides, both those for diagnostic purpose and those of the digital sets for the study, from a remote site, via secure access through a virtual private network and 2-factor authentication per institutional policy. Pathologists accessed their workstations located within the institutional enterprise firewall and had access to all electronic medical record. After all digital slides for diagnostic purpose were reviewed for each case, a complete final report was entered into the laboratory information system, but not electronically released to the medical record from the remote site.

***Statistics***

Stan runs a No U-Turn sampler, an extension to Hamiltonian Monte Carlo (HMC) sampling, a form of Markov Chain Monte Carlo (1-3). Four chains for 4000 iterations were generated. The final 2000 iterations of each chain converge as indicated by post-modelling diagnostics such as the number of effective samples and Gelman-Rubin $\hat{R}$ (4). A satisfactory posterior predictive model performance was ensured before using sample means (for estimates) and sample quantiles (for model’s compatibility intervals (CI)) (4,5). CI were calculated as 89% of the highest posterior density interval (HDPI). Predictive accuracy was measured through widely applicable information criteria (WAIC) and Pareto smoothed importance sampling approximate of the leave-one-out cross-validation (PSIS-LOO-CV) (6,7).

**SUPPLEMENTARY REFERENCES**

1. Metropolis N, Rosenbluth AW, Teller E, Et al. Equation of State Calculations by Fast Computing Machines. J. Chem. Phys. 1953;21:1087–1092.
2. Hoffman MD, Gelman A. The no-U-turn sampler: Adaptively setting path lengths in Hamiltonian Monte Carlo. J. Mach. Learn. Res. 2014;15:1593–1623.
3. Gelman A. et al. Bayesian data analysis. (CRC press, 2013).
4. McElreath R. Statistical rethinking: A Bayesian course with examples in R and Stan. (CRC press, 2020).
5. Gelman A. Discussion paper analysis of variance - Why it is more important than ever. Ann. Stat. 2005;33:1–53.
6. Watanabe S. Asymptotic equivalence of Bayes cross validation and widely applicable information criterion in singular learning theory. J. Mach. Learn. Res. 2010;11:3571–3594
7. Vehtari A, Gelman A, Gabry J. Practical Bayesian model evaluation using leave-one-out cross-validation and WAIC. Stat. Comput. 2017;27:1413–1432 Gelman A, Hwang J, Vehtari A. Understanding predictive information criteria for Bayesian models. Stat. Comput. 2014;24:997–1016

**SUPPLEMENTARY TABLES**

| **Table S1.** Prior predictive simulation | | | | |
| --- | --- | --- | --- | --- |
|  | mean | sd | 5.50% | 94.50% |
| a.1 | -0.01 | 1.01 | -1.65 | 1.58 |
| a.2 | 0.01 | 0.97 | -1.57 | 1.52 |
| a.3 | 0.04 | 1.02 | -1.56 | 1.64 |
| a.4 | -0.01 | 1 | -1.66 | 1.58 |
| a.5 | -0.01 | 1.03 | -1.59 | 1.65 |
| a.6 | 0.01 | 1.06 | -1.72 | 1.71 |
| a.7 | -0.01 | 1.05 | -1.69 | 1.69 |
| a.8 | -0.01 | 1.04 | -1.58 | 1.72 |
| a.9 | -0.03 | 1.04 | -1.7 | 1.66 |
| a.10 | 0.03 | 1.02 | -1.56 | 1.69 |
| a.11 | -0.01 | 1.01 | -1.59 | 1.61 |
| a.12 | -0.01 | 1.03 | -1.71 | 1.64 |
| a.13 | -0.01 | 1.05 | -1.59 | 1.71 |
| a.14 | 0.05 | 1.01 | -1.57 | 1.64 |
| a.15 | -0.03 | 0.99 | -1.63 | 1.61 |
| a.16 | 0 | 0.94 | -1.46 | 1.6 |
| a.17 | 0 | 0.99 | -1.62 | 1.63 |
| b.1 | -0.01 | 1.04 | -1.68 | 1.69 |
| b.2 | 0 | 1.03 | -1.65 | 1.66 |
| b.3 | -0.01 | 0.93 | -1.44 | 1.46 |
| b.4 | -0.04 | 0.97 | -1.6 | 1.46 |
| g.1 | -0.03 | 0.97 | -1.62 | 1.53 |
| g.2 | 0 | 0.94 | -1.45 | 1.5 |
| g.3 | -0.01 | 1 | -1.56 | 1.56 |
| g.4 | -0.02 | 1.02 | -1.6 | 1.7 |
| g.5 | -0.01 | 0.98 | -1.48 | 1.53 |
| d.1 | 0.01 | 1.07 | -1.85 | 1.67 |
| d.2 | -0.02 | 1.02 | -1.66 | 1.65 |
| e.1 | 0.02 | 1.04 | -1.67 | 1.63 |
| e.2 | -0.03 | 1.01 | -1.63 | 1.6 |
| e.3 | -0.02 | 0.98 | -1.54 | 1.55 |
| a_bar | -0.04 | 1.5 | -2.41 | 2.31 |
| sigma_a | 1.02 | 1.02 | 0.04 | 2.91 |
| sigma_b | 1.02 | 0.93 | 0.08 | 2.78 |
| sigma_g | 0.99 | 1.02 | 0.06 | 2.83 |
| sigma_d | 1.04 | 1.09 | 0.05 | 3.15 |
| sigma_e | 1.03 | 0.97 | 0.08 | 2.92 |

| **Table S2.** Estimates, standard errors, CI, effective samples, Rhat4 | | | | | |  |
| --- | --- | --- | --- | --- | --- | --- |
|  | mean | sd | 5.50% | 94.50% | n_eff | Rhat4 |
| a[1] | -0.21 | 0.62 | -1.21 | 0.77 | 2198 | 1 |
| a[2] | -0.32 | 0.63 | -1.35 | 0.68 | 2222 | 1 |
| a[3] | 0.21 | 0.61 | -0.75 | 1.23 | 1620 | 1 |
| a[4] | -1.06 | 0.75 | -2.25 | 0.12 | 2123 | 1 |
| a[5] | 0.04 | 0.65 | -1.03 | 1.04 | 1937 | 1 |
| a[6] | -0.11 | 0.67 | -1.2 | 0.96 | 2186 | 1 |
| a[7] | -0.91 | 0.7 | -2.04 | 0.16 | 2384 | 1 |
| a[8] | -0.68 | 0.67 | -1.77 | 0.34 | 2521 | 1 |
| a[9] | -0.18 | 0.65 | -1.25 | 0.84 | 1710 | 1 |
| a[10] | -0.66 | 0.66 | -1.71 | 0.31 | 2134 | 1 |
| a[11] | 0.1 | 0.63 | -0.96 | 1.08 | 1518 | 1 |
| a[12] | -1.04 | 0.69 | -2.18 | -0.01 | 1242 | 1 |
| a[13] | 2.01 | 0.64 | 1.05 | 3.09 | 1246 | 1 |
| a[14] | 0.28 | 0.64 | -0.76 | 1.23 | 1325 | 1 |
| a[15] | 0.97 | 0.57 | 0.12 | 1.91 | 1431 | 1 |
| a[16] | 0.62 | 0.59 | -0.31 | 1.57 | 1625 | 1 |
| a[17] | 0.21 | 0.64 | -0.79 | 1.26 | 1669 | 1 |
| b[1] | 0.52 | 0.89 | -0.87 | 1.97 | 1660 | 1 |
| b[2] | -0.41 | 0.82 | -1.66 | 0.89 | 1718 | 1 |
| b[3] | -0.39 | 0.79 | -1.63 | 0.93 | 1697 | 1 |
| b[4] | -0.07 | 0.89 | -1.46 | 1.39 | 2245 | 1 |
| g[1] | 0.16 | 0.51 | -0.62 | 0.98 | 954 | 1 |
| g[2] | -0.76 | 0.53 | -1.62 | 0.06 | 1257 | 1 |
| g[3] | -0.05 | 0.48 | -0.82 | 0.72 | 1017 | 1 |
| g[4] | -1.39 | 0.64 | -2.46 | -0.43 | 1360 | 1 |
| g[5] | 1.03 | 0.66 | 0.03 | 2.1 | 848 | 1 |
| d[1] | -1.01 | 0.68 | -2.11 | 0.02 | 1832 | 1 |
| d[2] | 0.19 | 0.79 | -1.02 | 1.54 | 1447 | 1 |
| e[1] | 0.64 | 0.76 | -0.49 | 1.88 | 1114 | 1 |
| e[2] | -0.28 | 0.61 | -1.22 | 0.71 | 1444 | 1 |
| e[3] | -1.02 | 0.66 | -2.1 | 0.02 | 1749 | 1 |
| a_bar | -1.77 | 1.08 | -3.35 | 0.04 | 1173 | 1 |
| sigma_a | 0.74 | 0.21 | 0.46 | 1.1 | 942 | 1 |
| sigma_b | 0.43 | 0.39 | 0.04 | 1.13 | 1022 | 1 |
| sigma_g | 1.16 | 0.51 | 0.58 | 2.15 | 927 | 1 |
| sigma_d | 1.23 | 0.81 | 0.39 | 2.75 | 1233 | 1 |
| sigma_e | 0.89 | 0.58 | 0.31 | 1.94 | 1011 | 1 |

| **Supplementary table 3.** Estimate of out-of-sample relative K-L divergence | | | | |
| --- | --- | --- | --- | --- |
|  | estimate | lppd | penalty | std_err |
| WAIC | 689.90 | -325.32 | 19.63 | 44.53 |
| PSIS | 690.14 | -345.07 | 19.75 | 44.57 |
