# Supplementary figures and images for: Digital Pathology During the COVID-19 Outbreak in Italy: Survey Study
Source: J Med Internet Res. 2021 Feb 22;23(2):e24266. doi: 10.2196/24266 (PMC7901595; doi:10.2196/24266)

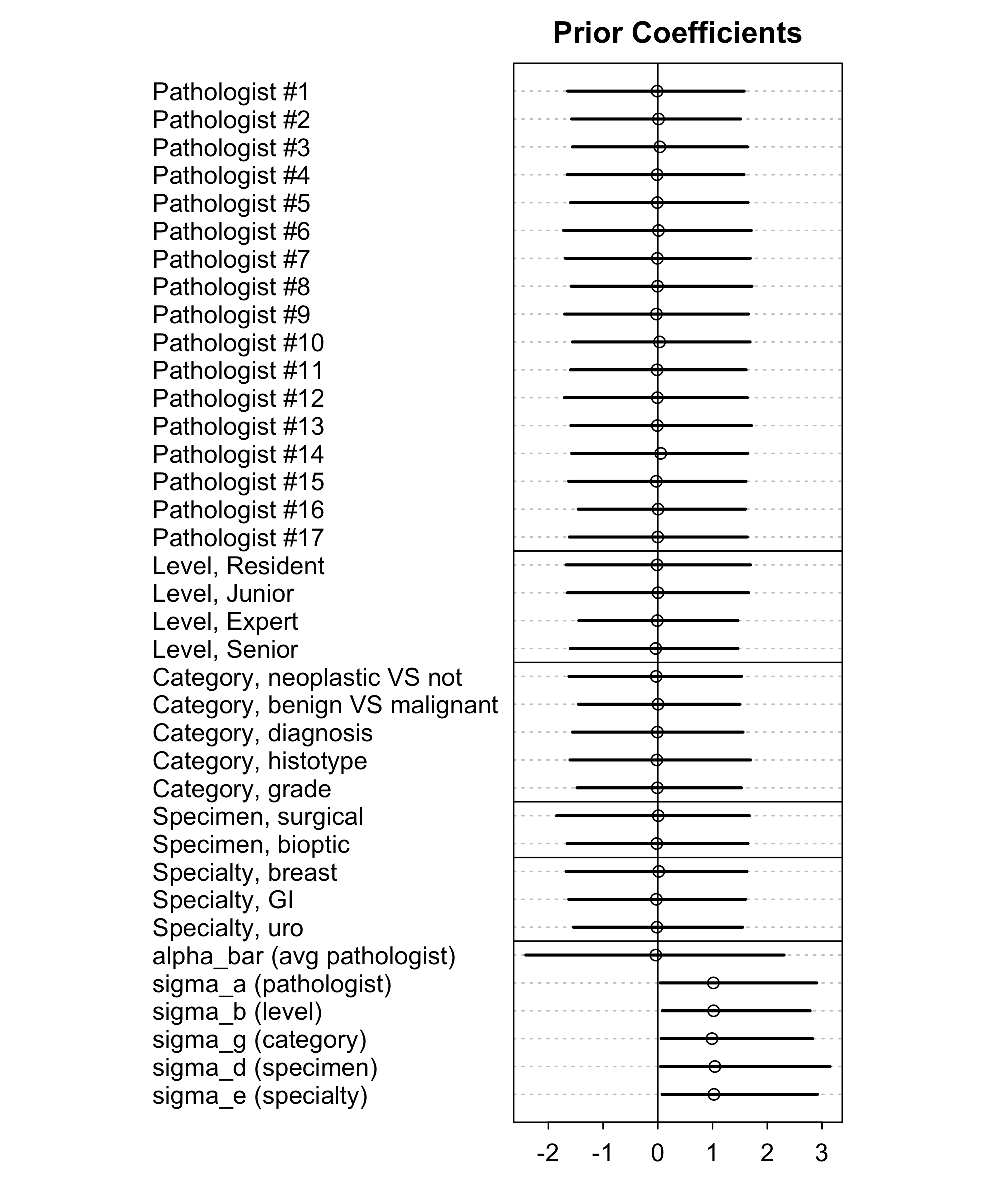

Supplement: Multimedia Appendix 2 [file jmir_v23i2e24266_app2.png]

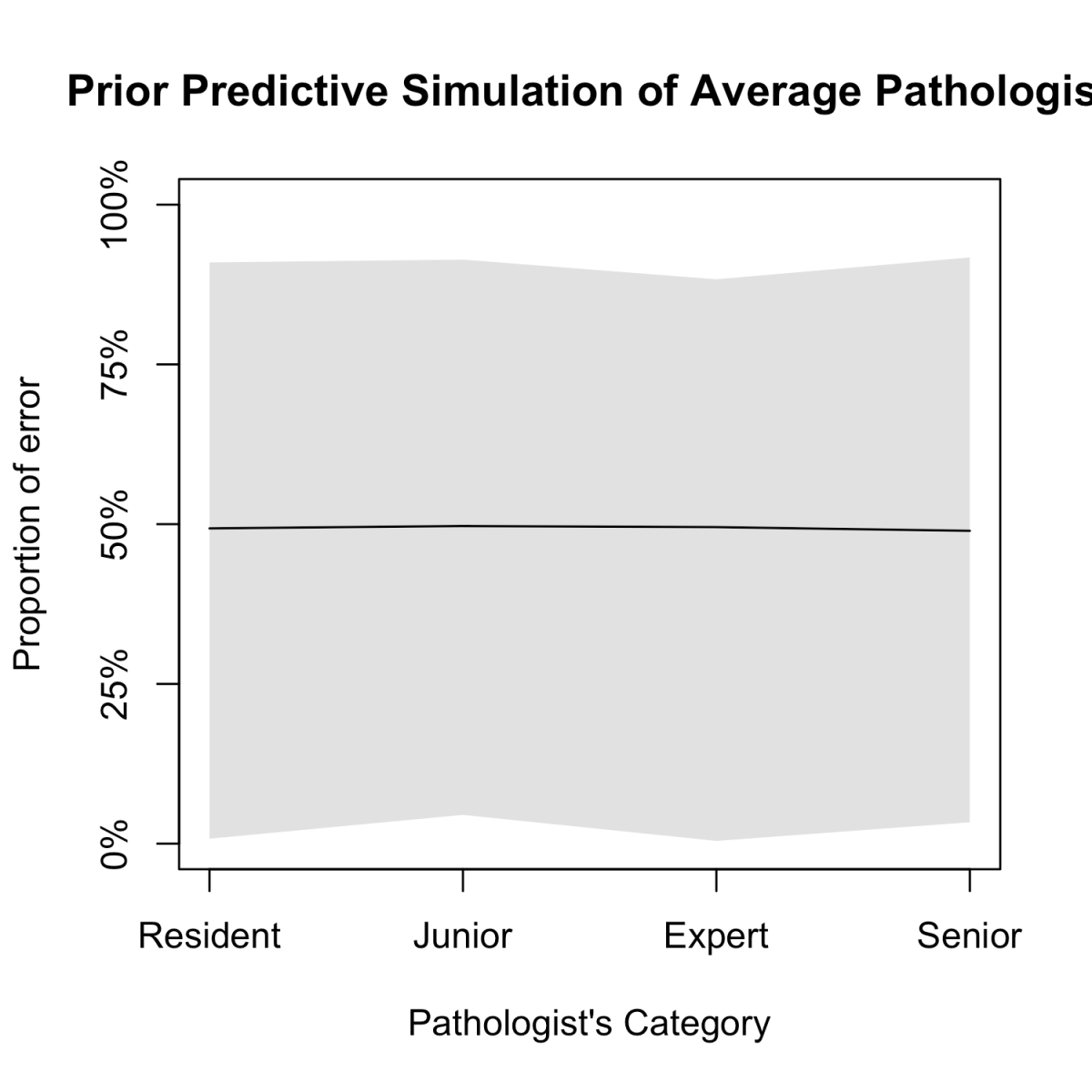

Supplement: Multimedia Appendix 3 [file jmir_v23i2e24266_app3.png]

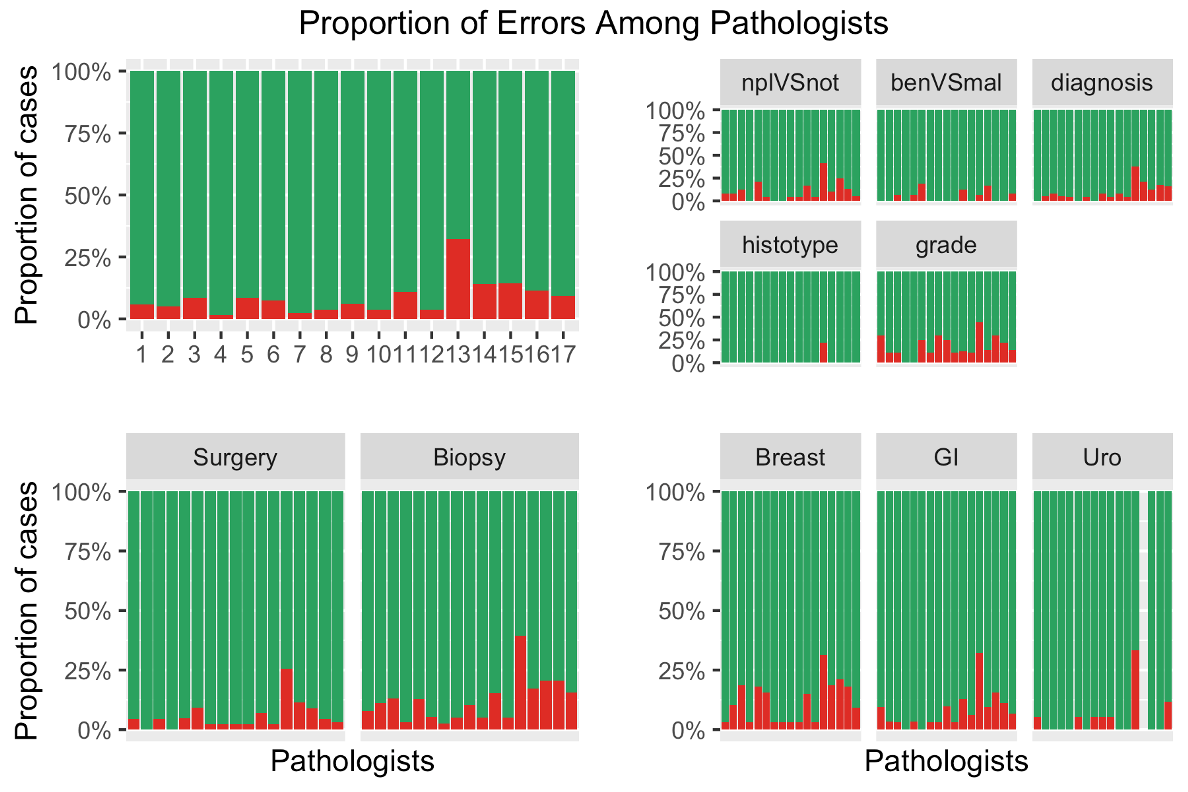

Supplement: Multimedia Appendix 4 [file jmir_v23i2e24266_app4.png]

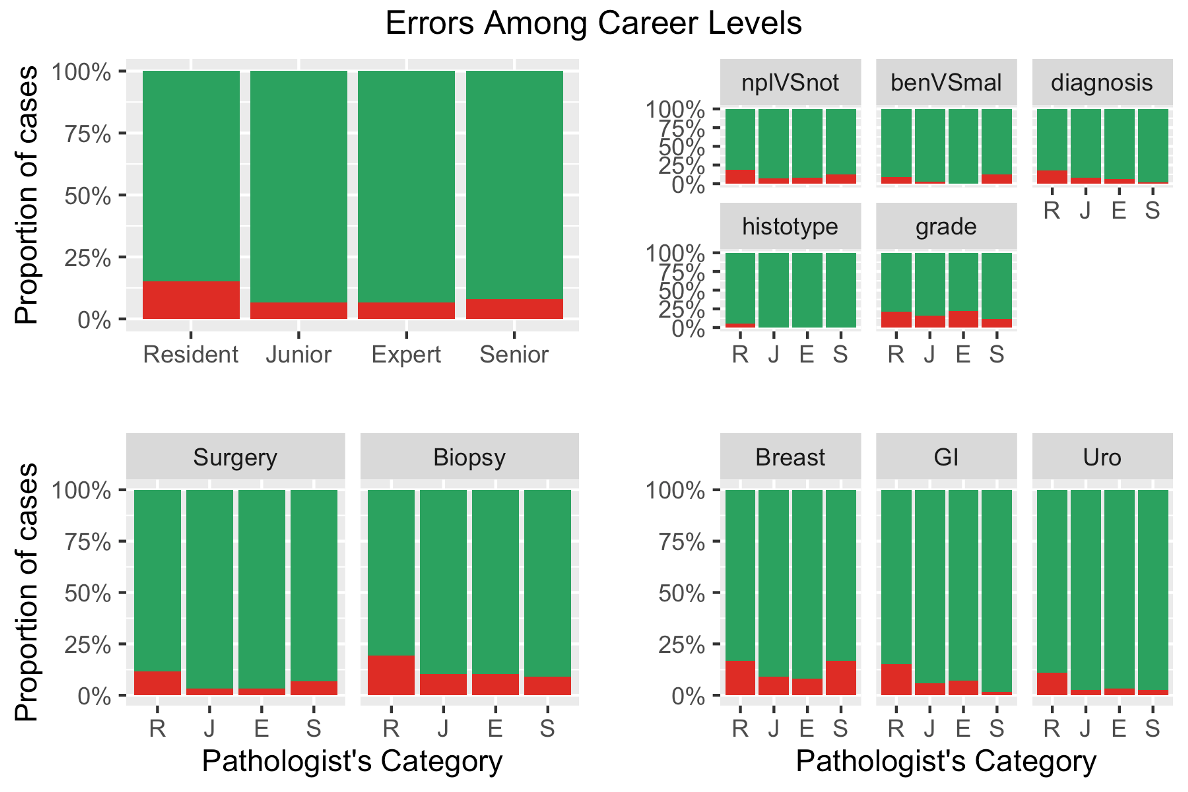

Supplement: Multimedia Appendix 5 [file jmir_v23i2e24266_app5.png]

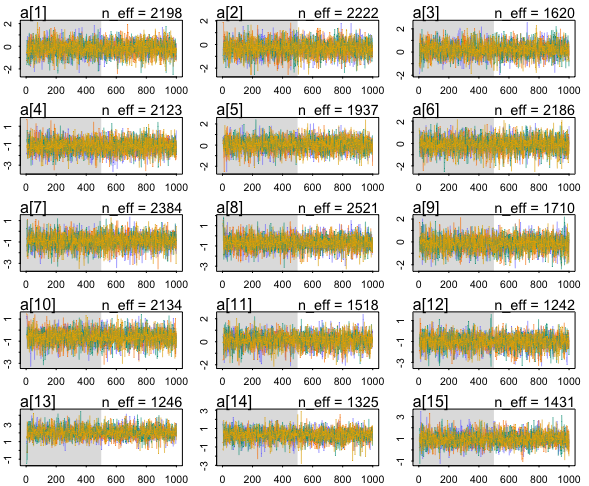

Supplement: Multimedia Appendix 6 [file jmir_v23i2e24266_app6.png]

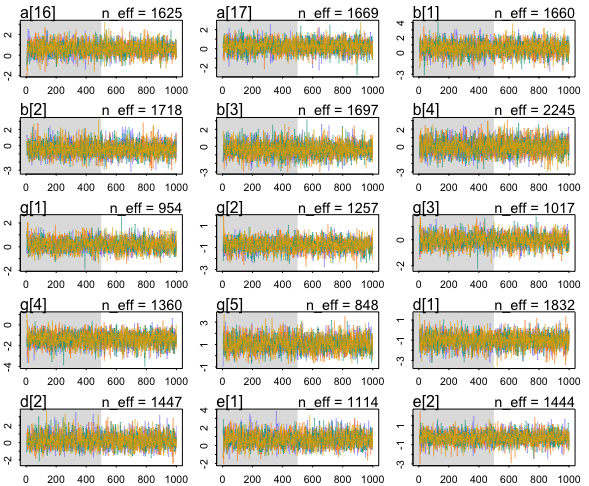

Supplement: Multimedia Appendix 7 [file jmir_v23i2e24266_app7.png]

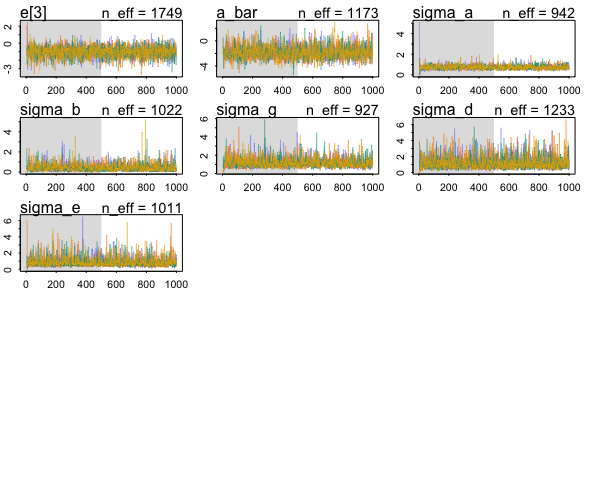

Supplement: Multimedia Appendix 8 [file jmir_v23i2e24266_app8.png]

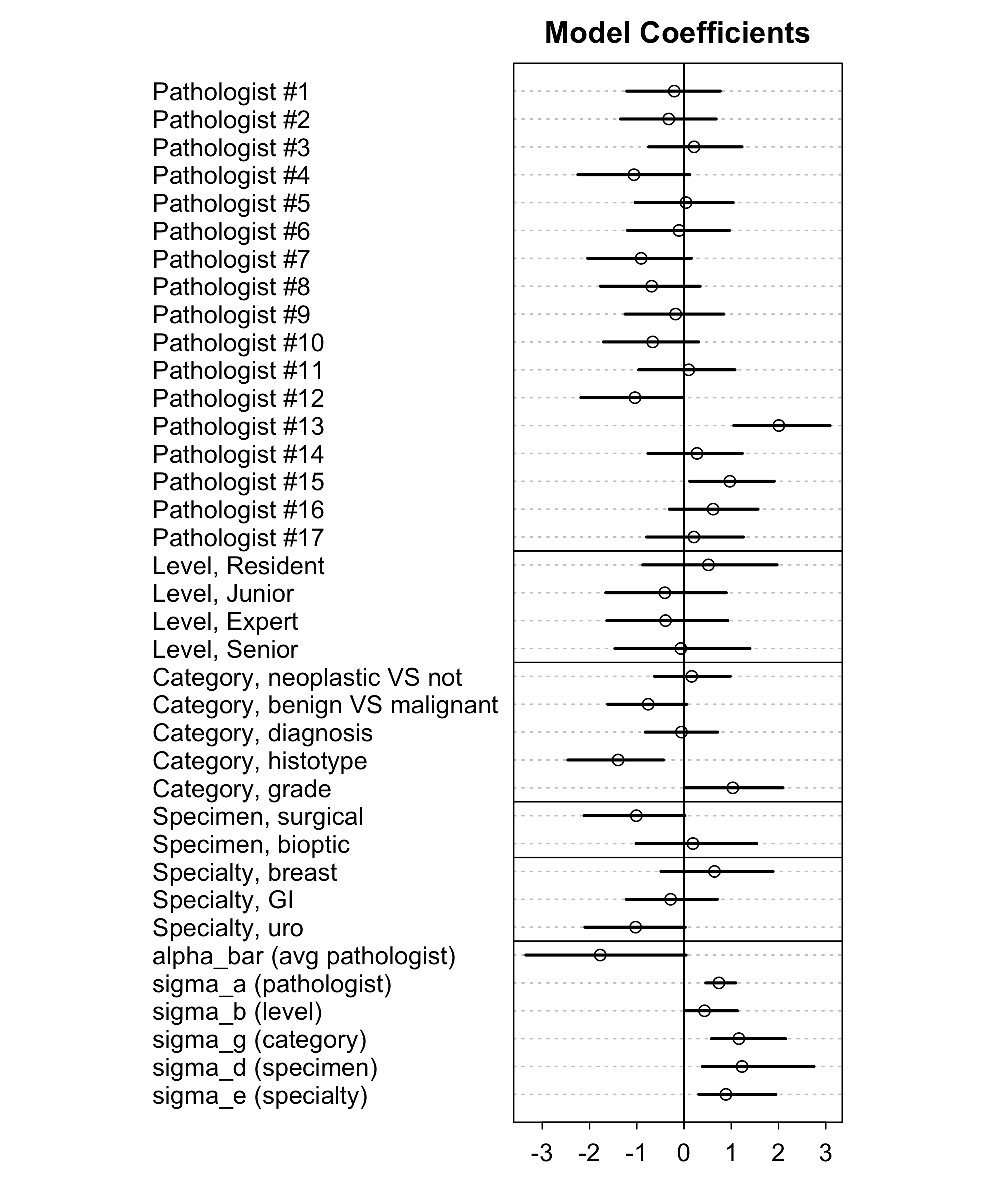

Supplement: Multimedia Appendix 9 [file jmir_v23i2e24266_app9.png]
